# Commissioning a multileaf collimator virtual cone for the stereotactic radiosurgery of trigeminal neuralgia

**DOI:** 10.1002/acm2.13562

**Published:** 2022-02-14

**Authors:** Thomas A. D. Brown, Rex G. Ayers, Richard A. Popple

**Affiliations:** ^1^ Northwest Medical Physics Center Lynnwood Washington USA; ^2^ Department of Radiation Oncology The University of Alabama at Birmingham Birmingham Alabama USA

**Keywords:** stereotactic radiosurgery, trigeminal neuralgia, virtual cone

## Abstract

A multileaf collimator (MLC), virtual‐cone treatment technique has been commissioned for trigeminal neuralgia (TGN) at Tri‐Cities Cancer Center (TCCC). This novel technique was initially developed at the University of Alabama in Birmingham (UAB); it is designed to produce a spherical dose profile similar to a fixed, 5‐mm conical collimator distribution. Treatment is delivered with a 10‐MV flattening‐filter‐free (FFF) beam using a high‐definition MLC on a Varian Edge linear accelerator. Absolute dose output and profile measurements were performed in a 20 × 20 × 14 cm^3^ solid‐water phantom using an Exradin W2 scintillation detector and Gafchromic EBT3 film. Dose output constancy for the virtual cone was evaluated over 6 months using an Exradin A11 parallel plate chamber. The photo‐neutron dose generated by these treatments was assessed at distances of 50 and 100 cm from isocenter using a Ludlum Model 30–7 Series Neutron Meter. TGN treatments at TCCC have been previously delivered at 6‐MV FFF using a 5‐mm stereotactic cone. To assess the dosimetric impact of using a virtual cone, eight patients previously treated for TGN with a 5‐mm cone were re‐planned using a virtual cone. Seven patients have now been treated for TGN using a virtual cone at TCCC. Patient‐specific quality assurance was performed for each patient using Gafchromic EBT‐XD film inside a Standard Imaging Stereotactic Dose Verification Phantom. The commissioning results demonstrate that the virtual‐cone dosimetry, first described at UAB, is reproducible on a second Edge linear accelerator at an independent clinical site. The virtual cone is a credible alternative to a physical, stereotactic cone for the treatment of TGN at TCCC.

## 1 INTRODUCTION

A multileaf collimator (MLC), virtual‐cone technique has been developed for the treatment of trigeminal neuralgia (TGN) and essential tremors at the University of Alabama in Birmingham (UAB).^1^ This technique utilizes the 120‐leaf, high‐definition MLC (HD120 MLC) on a Varian Edge linear accelerator (Varian Medical Systems, Palo Alto, CA) to produce a spherical dose distribution that approximates the distribution of a fixed, 5‐mm stereotactic cone. The HD120 MLC is comprised of 32 central leaf pairs with a width of 2.5 mm and 28 outer pairs with a width of 5 mm. The virtual‐cone field size, 2.1 × 5 mm^2^, is defined by the two central collimator leaf pairs, whose position remains fixed for treatment. Treatment is delivered with a 10‐MV flattening filter‐free (FFF) beam using either 10 (TGN) or 20 (essential tremors) non‐coplanar, parasagittal arcs; the monitor units (MU) delivered per degree of rotation is varied as the sine of the gantry angle. A beam energy of 10‐MV FFF is used to exploit a higher dose rate of 2400 MU/min, compared with 1400 MU/min for 6‐MV FFF, thereby reducing the treatment time where the dose is as high as 150 Gy in a single fraction.^2^

The virtual cone potentially offers an alternative to physical, stereotactic cones for linac‐based treatments of TGN and essential tremors. Stereotactic cones impose an additional financial cost to a new stereotactic radiosurgery (SRS) program and increase the treatment and quality assurance (QA) burden for therapists and physicists, especially in a community‐practice setting where staffing resources can be limited. Although there is the potential for large changes in beam output with small changes in leaf gap,^3^ virtual‐cone commissioning data obtained at UAB showed promising output constancy.^1^ This work also described a simple method for adjusting the treatment planning model of the HD120 MLC to replicate the dosimetry of these treatments. However, at this time, the virtual‐cone technique has not been reproduced at a second clinical site, and published commissioning and treatment data are limited to a single linac.

Tri‐Cities Cancer Center (TCCC) has collaborated with UAB to commission the virtual cone as a potential alternative to a physical, 5‐mm stereotactic cone for local treatments of TGN. TCCC is a nonprofit, community‐based cancer center that utilizes a Varian Edge for SRS treatments with HD120 MLC and stereotactic cones. All SRS treatment planning is performed in Eclipse (Varian Medical Systems). Patients are immobilized for SRS on a six‐degree‐of‐freedom robotic couch using a Qfix Encompass mask (Qfix, Avondale, PA, USA). Cone‐beam computed tomography (CBCT) is used for patient positioning in all SRS treatments. At least two CBCTs are performed for each patient; a second CBCT is used to verify the patient has not slipped inside the mask following pitch and roll adjustments after the first CBCT. An AlignRT optical surface monitoring system (VisionRT Ltd, London, UK) is used for real‐time patient monitoring and beam control. Monitoring tolerances of 1 mm and 0.5^o^ are used during treatment. The AlignRT system has demonstrated sub‐millimeter precision for non‐coplanar SRS treatments.^4^, ^5^ TGN has been treated at TCCC using a 5‐mm diameter, stereotactic cone for prescriptions of 70–90 Gy to the maximum dose in a single fraction.^6^ Treatment is delivered with a 6‐MV FFF beam to the root of the affected trigeminal nerve using five non‐coplanar, parasagittal arcs. To date, 53 patients have been treated at TCCC for TGN using this method.

Prior to commissioning the virtual cone, the TCCC Edge had been commissioned for standard MLC‐based, SRS treatments at 6‐ and 10‐MV FFF consistent with published recommendations.^7^, ^8^ Output factors for jaw sizes down to 1 × 1 cm^2^ have been measured using a Sun Nuclear EDGE detector (Sun Nuclear, Melbourne, FL, USA). However, output factors for jaw sizes < 3 × 3 cm^2^ do not affect Eclipse calculation results for small MLC‐collimated fields in machines where the MLC is located below the jaws.^9^ Small‐field correction factors were not applied to these measurements; the small‐field correction factor at 6‐ and 10‐MV FFF for a jaw size of 3 × 3 cm^2^ is 0.999.^10^ The geometric accuracy of SRS treatments is evaluated using a Winston‐Lutz test. Day‐of‐treatment tests for the HD120 MLC have demonstrated an isocenter‐target coincidence of less than 0.6 mm over the full range of gantry, collimator, and couch rotation. The isocenter‐target coincidence is slightly smaller for stereotactic cones (<0.5 mm). This is partly the result of excluding the effect of collimator rotation since it is never performed for patient treatments with cones. A successful, independent end‐to‐end test at 10‐MV FFF was obtained using the Imaging and Radiation Oncology Core Houston SRS head phantom for an HD120 MLC treatment.^11^ Thermoluminescent dosimeter measurements showed agreement with the planned dose for a 1.9‐cm target to within 1%; film results showed a passing rate of 100% in the coronal and sagittal planes for gamma‐index criteria of 5% and 3 mm.^12^

This paper describes the measurements and QA steps used for the streamlined commissioning of a virtual cone at TCCC. These commissioning data are benchmarked against the UAB results. We demonstrate the feasibility of replicating this technique in a community‐practice setting and present a comparison of the virtual cone with a stereotactic cone for the treatment of TGN.

## 2 METHODS AND MATERIALS

### 2.1 Treatment planning script and beam model

An executable Eclipse script,^1^ provided by UAB, was used to generate the treatment fields used for virtual‐cone planning and treatment at 10‐MV FFF. The script generates an identical beam geometry for every treatment plan. Ten dynamic arcs are generated for five unique couch positions: two arcs per couch angle, where the collimator is set to 45° for one arc and 135° for the other arc. Couch angles 0, 36, 72, 288, and 324^o^ are used for treatment. Two 360^o^ arcs are used at a couch angle of 0^o^ and partial arcs of 180^o^ are used for the remaining couch angles. The field size for each arc is defined by the two central HD120 MLC leaf pairs. These leaves have a width of 2.5 mm and their positions are fixed so that the physical gap between both opposing pairs is 2.1 mm. The other HD120 MLC leaves are moved to positions outside the jaw size which is fixed at 1.5 × 1.5 cm^2^. For each arc, the number of MU delivered per degree of rotation is proportional to the sine of the gantry angle; planning control points are defined every two degrees. The field parameters used in this script are designed to generate a spherical dose distribution with an equivalent diameter of approximately 5 mm.^1^ Dose calculations are performed for a 1‐mm dose grid using the Analytical Anisotropic Algorithm (AAA).

The physical leaf gap of 2.1 mm was determined specifically for the TCCC Edge. The dosimetric properties of the HD120 MLC leaves are modeled in Eclipse using transmission and dosimetric leaf gap (DLG) values for each beam energy. Replicating the virtual cone developed at UAB requires consideration of the measured DLG. Popple et al.^1^ determined that for a 10‐MV FFF DLG value of 0.36 mm, a physical leaf gap of 2.1 mm yielded a dose distribution profile with a 50% isodose equivalent diameter of 5 mm. To reproduce the same dosimetric profile on the TCCC Edge, the physical gap should be set to 2.1 mm + 0.36 mm ‐ measured DLG (mm). DLG and transmission values are verified annually at TCCC. The DLG is extrapolated from output measurements of seven sweeping leaf gaps with widths varying from 2–20 mm.^3^ DLG and transmission measurements are performed at a depth of 10 cm in a solid‐water phantom for a source‐to‐detector distance (SDD) of 100 cm using a Farmer chamber. The most recent DLG measurement for 10‐MV FFF, prior to this work, yielded a value of 0.43 ± 0.05 mm. The uncertainty in this measurement is the standard deviation of the seven most recent DLG measurements acquired at this energy. Any systematic error associated with this measurement was assumed to be small in comparison. This result indicates that a physical gap of 2.03 ± 0.05 mm should be used for the TCCC Edge; however, since this is less than 0.1 mm (2σ) different from the UAB value, a physical gap of 2.1 mm was retained for the sake of consistency.

A separate machine model was created in Eclipse for virtual‐cone treatments. Although DLG values have been measured for each beam energy at TCCC, these values were changed for the beam model during the commissioning process described by AAPM Task Group 119.^13^ This modification was performed to improve the agreement between measurement and calculated dose for intensity‐modulated radiation therapy (IMRT) treatments. For this work, a new machine model was created for 10‐MV FFF only. The new model was calculated from the same beam data as the old model except for the DLG and transmission values which were changed to match the most recent measurements (0.43 ± 0.05 mm, 1.43 ± 0.07%). The new machine model was not designated as an equivalent machine to the Edge in Eclipse; therefore, a machine override was required at the treatment console prior to beam delivery. The override provides an important QA check prior to treatment.

### 2.2 Output and profile verification

The virtual‐cone field size is fixed at 2.1 × 5 mm^2^. Given this small size, the dose output and profile of these treatments are critically dependent on the position of the HD120 MLC leaves. To ensure a reproducible HD120 MLC gap of 2.1 mm for commissioning, routine QA, and patient treatments, the HD120 MLC was re‐initialized on each day of measurement and treatment. In addition to re‐initialization, the HD120 MLC leaves were fully retracted immediately prior to beam on and then driven back to their plan values. This action has the effect of resetting the HD120 MLC leaf positions, which may deviate from their plan values under the action of gravity as the gantry is rotated into position for treatment.^14^

The dose output and profile of a virtual‐cone treatment were verified using an Exradin W2 scintillation detector (Standard Imaging, Middleton, WI, USA) and Gafchromic EBT3 film (Ashland Advanced Materials, Bridgewater, NJ, USA). The W2 detector was used to verify the output for a plan designed to deliver 90 Gy; the typical dose used for TGN treatments at TCCC. The film was used to verify the dose output and profile in the coronal plane for a plan designed to deliver 5 Gy. A lower dose had to be used for the film measurement since EBT3 film was the only film type available to the clinic at that time; its dynamic range is limited to doses below 20 Gy and is most accurate for doses below 10 Gy.^15^ Both treatment plans were designed on a 20 × 20 × 14 cm^3^ virtual‐water phantom in Eclipse. For the 90‐Gy plan, isocenter was placed at the center of the phantom. For the 5‐Gy plan, isocenter was offset from the phantom center by 1 cm and placed at a depth of 6 cm. The dose was calculated using the AAA v15.6 algorithm for a 1‐mm dose grid. The dose was scaled so that the prescription dose was equal to the maximum calculated dose.

The W2 detector measurement was performed at the center of a 20 × 20 × 14 cm^3^ solid‐water phantom. The phantom was aligned to isocenter using AP and lateral kV imaging fields. A dummy detector fiber containing a small ball bearing at the distal tip was inserted into the phantom and used as a landmark for alignment with isocenter. The detector was calibrated for dose in this phantom using a static, 4 × 4 cm^2^ field designed to deliver 2 Gy to isocenter. The MU for this calibration field was calculated in Eclipse on the virtual‐water phantom. Two W2 output measurements were performed, set up independently on separate days, to verify the reproducibility of this technique. The EBT3 film measurement was performed in the same phantom. A piece of film measuring approximately 5 × 5 cm^2^ was placed on the central axis of the phantom at a depth of 6 cm. The phantom was aligned to isocenter using the treatment room lasers for the film measurement.

The EBT3 film was calibrated for dose by irradiating film, taken from the same manufacturing lot number, under reference conditions in a solid‐water phantom. Known doses determined from an AAPM Task‐Group 51 dose calibration^16^, ^17^ were delivered at 6 MV to calibration film placed at d_max_ using a 10 × 10 cm^2^ field size and a source‐to‐film distance of 100 cm.

The film used to measure the virtual‐cone treatment was digitized 24 hours after irradiation using an Epson Perfection V750 Pro flatbed scanner (Epson America, Los Alamitos, CA, USA). A cardboard template was used to align the film so that it was centered on the scanner bed and the film was scanned in the portrait orientation, consistent with the film used for dose calibration. The film images were saved as 24‐bit RGB TIF files at a resolution of 0.08 mm per pixel and processed using DoseLab (Varian Medical Systems). The RGB files were converted into optical density using the red channel. A third‐order polynomial fitted to seven calibration points (*r* ^2^ = 0.999 for 0–6 Gy) was used to convert the optical density into dose. For the purposes of reducing the noise in the film images, each image was smoothed in DoseLab using a disk average algorithm. This algorithm averaged the pixels over a circular disk with a radius of 5 pixels.

The W2 and film measurements were performed over 2 days. The machine output was not verified with a primary dosimeter, such as a Farmer chamber, on either of these days. However, a Task‐Group 51 dose calibration was performed for the TCCC Edge 1 week after these measurements, where the 10‐MV FFF output was determined to be within 0.1% of the expected value. Daily output measurements performed with a DailyQA3 device (Sun Nuclear) show there was no change greater than 0.5% in the 10‐MV FFF output over the time period between the W2/film measurements and the dose calibration.

### 2.3 Output factor constancy

The output constancy of the virtual‐cone treatments was evaluated using the same method developed by Popple et al.^1^ The average output of two static fields with the same HD120 MLC gap (2.1 mm) and collimation rotation settings (45 and 135^o^) as the virtual cone was evaluated using an Exradin A11 parallel‐plate chamber. Measurements were performed in solid water at a depth of 2.3 cm for an SDD of 100 cm. The output was characterized relative to the output from a beam with a 10 × 10 cm^2^ field size. The output factor constancy was evaluated for fifteen measurements performed over a time period of 6 months. The sensitivity of this technique was verified by performing output measurements for fields with an HD120 MLC gap of 2.2–2.4 mm and comparing these results with the output for a gap of 2.1 mm.

### 2.4 Neutron dose measurements

A virtual‐cone TGN treatment designed to deliver 90 Gy to the maximum dose requires approximately 30 000 MU. Although the photo‐neutron fluence is small for a beam energy of 10‐MV FFF,^18^ this MU value is an order of magnitude greater than the numbers used for other 10‐MV FFF treatments at TCCC. In addition, the jaws and HD120 MLC can be a significant source of neutrons if they block a large portion of the beam.^18^, ^19^

Neutron dose measurements were performed using a Ludlum Model 30–7 Series Neutron Meter (Ludlum Measurements Inc., Sweetwater, TX, USA) for a virtual‐cone treatment designed to deliver 90 Gy. Most neutron dose to the patient is the result of fast neutrons; energy measurements of secondary neutron spectra show a fast neutron peak centered between 0.1 and 1 MeV, a maximum energy of approximately 10 MeV, and a low‐energy tail arising from elastic scattering.^18^, ^19^ The Ludlum meter comprises a 19.5‐cm diameter REM ball containing a ^3^He detector. The energy response of the meter approximates the neutron dose equivalence per fluence from thermal energies to 7 MeV and can detect neutrons as high as 12 MeV.^20^ The neutron meter was placed on the patient couch for measurements performed at distances of 50 and 100 cm from isocenter. A 20 × 20 × 10 cm^3^ solid water phantom was placed at isocenter to simulate the head of the patient. The neutron meter has a gamma rejection specification of <10 cpm up to 10 R/h. Prior to these neutron measurements, a lnovision 451P Ion Chamber Survey Meter (Fluke Biomedical, Cleveland, OH, USA) was used to verify that the photon dose rate did not exceed 10 R/h at a distance of 50 cm.

### 2.5 TGN treatments

TGN treatments at TCCC have been delivered at 6‐MV FFF using a 5‐mm stereotactic cone. These treatments are comprised of five non‐coplanar, parasagittal arcs whose isocenter is placed on the root of the affected trigeminal nerve, approximately 3–4 mm from the brainstem. The exact location of isocenter is determined so that the dose constraints for the brainstem, the primary limiting critical structure, are satisfied (see Table 5). Unlike the virtual‐cone treatments, the beam geometry is adjusted for each patient. Each arc varies in length from approximately 90–180^o^; the beginning and end of each arc, as well as the corresponding couch angle, are chosen to avoid entrance and exit dose to the spinal cord, optic apparatus, and inner ears. The selection of couch angles is also determined by the laterality of the affected trigeminal nerve. For the purposes of reducing the beam path length and hence the number of MU, the couch angles for right‐sided treatments are concentrated between 0 and 90^o^ while the couch angles for left‐sided treatments are concentrated between 0 and 270^o^. Dose calculations for cones in Eclipse are performed for a 1‐mm dose grid using the Cone Dose Calculation algorithm. The planning CT slice thickness for these treatments is 0.625 mm.

To understand the dosimetric impacts of treating TGN with a virtual cone, eight patients previously treated for TGN with a stereotactic cone were re‐planned using the virtual‐cone script. The dose prescription and the location of isocenter were not changed for the virtual‐cone plans. The resulting dose distribution, including doses to the brainstem, optic apparatus, inner ears, and brain tissue, were compared between the two types of plan for each patient.

Seven patients have now been treated for TGN using a virtual cone. For each patient, a dose of 90 Gy was prescribed to the maximum dose in a single fraction. Patient‐specific QA was performed for each virtual‐cone plan using Gafchromic EBT‐XD film; this film type has a much wider dynamic range than EBT3 film and is far more appropriate for measuring stereotactic doses.^21^ Dose measurements in the coronal plane were performed using a 5 × 5 cm^2^ piece of film placed inside a Standard Imaging Stereotactic Dose Verification Phantom at a depth of 6 cm. A dose measurement in the sagittal plane, as well as the coronal plane, was performed for one of the patients. Unlike the coronal‐plane measurements, which were performed in a homogeneous, water‐equivalent medium, the sagittal plane measurement was performed with the phantom configured to contain five heterogeneity inserts with CT densities ranging from air to bone.

The EBT‐XD film was calibrated at 10‐MV FFF using the same method as the EBT3 film (Section 2.2). The film was also scanned and processed using the same procedure, although the EBT‐XD film images were converted into optical density using the green channel rather than the red. A third‐order polynomial fitted to ten calibration points (*r* ^2^ = 0.999 for 0–140 Gy) was used to convert optical density into dose. These QA measurements provided a final verification of the dose output and profile before patient treatment.

Table 1 provides an overview of all the detectors used for commissioning the virtual cone at TCCC.

**TABLE 1:** Summary of detector systems used for commissioning the virtual cone at Tri‐Cities Cancer Center (TCCC)

**Commissioning process**	**Measurement type**	**Detector system**
Beam model adjustment	HD120 MLC dosimetric leaf gap measurements	Farmer chamber
Output and profile verification	Virtual cone output	W2, EBT3 film
	Virtual cone profile	EBT3 film
	Absolute dose calibration	ADCL‐calibrated Farmer chamber
	Daily output constancy	DailyQA3
Output factor constancy	Virtual cone output factors	Exradin A11 parallel‐plate chamber
Neutron measurements	Virtual cone neutron dose	Ludlum 30–7 neutron meter
	Photon background	Inovision 451P survey meter
Patient‐specific QA	Virtual cone output and profile	EBT‐XD film

## 3 RESULTS AND DISCUSSION

### 3.1 Output and profile verification

#### 3.1.1 W2 detector measurements

**FIGURE 1:**
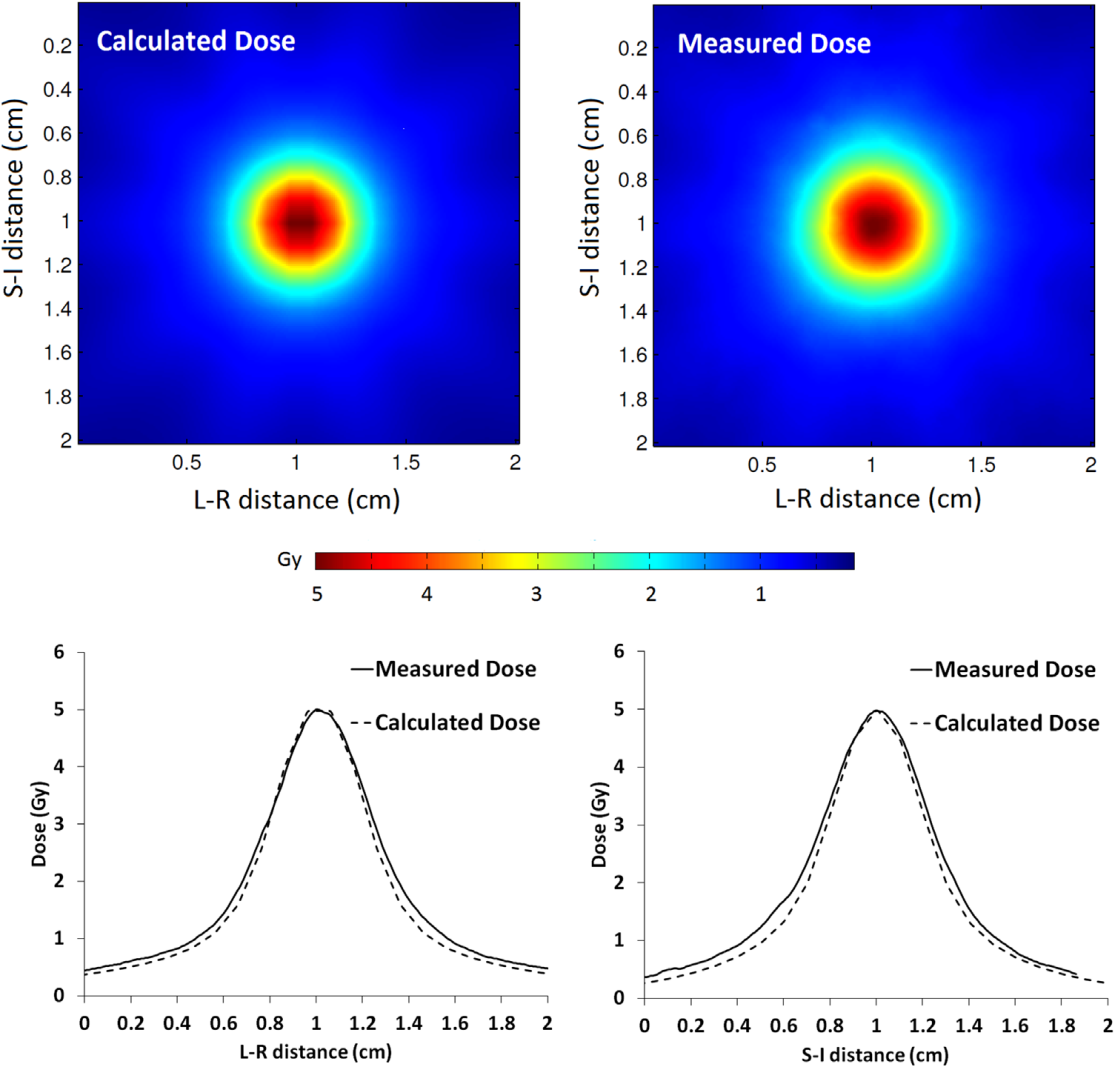
Measured (EBT3 film) and calculated (AAA v15.6) dose distributions in the coronal plane for a virtual cone designed to deliver 5 Gy. The dose distributions were registered in DoseLab for gamma analysis using the center of the distributions. The left‐right (L‐R) and superior‐inferior (S‐I) dose profiles are shown for comparison

The two W2 detector measurements yielded dose values of 87.23 ± 2.79 and 87.20 ± 2.79 Gy for a virtual cone designed to deliver 90 Gy. An uncertainty budget for the W2 detector measurements is shown in Table 2. The measured doses show good agreement with the planned dose: the average measured‐to‐plan dose ratio is 0.969 ± 0.023. Although not part of this work, W2 measurements performed at UAB on an Edge linac using an identical setup, including the same equipment and virtual‐cone configuration, have yielded a measured‐to‐plan dose ratio of 0.962. The small‐field correction factor reported for the W2 detector is 1.0 for field sizes as small as 4 mm.^22^ There is no correction data for field sizes as small as 2.1× 5 mm^2^; however, this is partly mitigated by the size of the dose distribution produced by the virtual cone (see Figures 1 and 3).

**TABLE 2:** Estimated uncertainty budget for W2 dose measurements. The largest source of uncertainty is associated with the detector position due to an initial dose fall‐off of approximately 10% per mm from isocenter. The uncertainty associated with the Task Group‐51 dose calibration for the linac^17^ is not included since it is a systematic error common to all dosimetric measurements and treatment planning calculations

**Dose measurement uncertainties (*k* = 1) for W2 detector**		
**Type of uncertainty**	**Source of uncertainty**	**Effect**
Statistical	Detector position	±3%
	Beam output	±0.25%
Systematic	Dose calibration (Eclipse TPR calculation for 4 × 4 cm^2^ field)	±0.5%
	Measurement in solid water^27^	±1%
Total		±3.2%

#### 3.1.2 EBT3 film measurements

Figure 1 shows the measured and calculated dose distributions in the coronal plane for a virtual cone designed to deliver 5 Gy. The calculated dose distribution was determined from a DICOM dose plane coincident with the expected position of the film in the phantom. The measured dose distribution was registered to this dose plane using the center of the dose distribution. A gamma analysis was applied to a 2 × 2 cm^2^ region of interest centered on the measured dose distribution. The EBT3 film measurement showed a gamma passing rate of 99.4% for an absolute dose difference of 2% and distance‐to‐agreement of 1 mm with a 10% threshold. The global maximum was used as the reference dose for dose difference normalization.

The film measurement yielded a maximum dose of 5.03 ± 0.22 Gy. An uncertainty budget for the film measurements is shown in Table 3. The 50% and 25% dose widths are shown in Table 4. The calculated widths are slightly larger than the calculated widths reported by Popple et al.^1^ for an anthropomorphic skull phantom, but they agree to within 0.3 mm at 50% and 0.8 mm at 25%. The differences between the calculated and measured widths shown in Table 4 are significant in the context of their uncertainties, but they indicate a distance‐to‐agreement of <0.7 mm for isodose lines greater than 25%.

**TABLE 3:** Estimated uncertainty budget for EBT3 and EBT‐XD film measurements. One of the largest sources of uncertainty is associated with film position due to an initial dose fall‐off of approximately 10% per mm from isocenter. The uncertainty associated with the Task Group‐51 dose calibration for the linac^17^ is not included since it is a systematic error common to all dosimetric measurements and treatment planning calculations. The EBT‐XD film measurements were acquired over a time period of 6 months; a larger beam output uncertainty, relative to the EBT3 film measurement, was assigned to these measurements

**Measurement uncertainties (*k* = 1) for EBT3 & EBT‐XD Film**		
**Maximum dose uncertainties**		
**Type of uncertainty**	**Source of uncertainty**	**Dose effect**
Statistical	Film position	±3%
	Beam output	±0.25% (EBT3 film), ±0.5% (EBT‐XD Film)
Systematic	Film calibration and dose extraction from red (EBT3)/green (EBT‐XD) channel^28^, ^29^	±3%
	Correction for film image resolution and smoothing	±0.3%
Total		±4.3% (EBT3 film), ±4.3% (EBT‐XD film)

**Dose width uncertainties**		
**Type of uncertainty**	**Source of uncertainty**	**Width effect**
Statistical	Film position	±1%
Systematic	Correction for film image resolution and smoothing	±1%
Total		±1.4%

**TABLE 4:** Measured (EBT3 film) and calculated (AAA v15.6) dose widths for a virtual cone designed to deliver 5 Gy. The dose widths were determined from orthogonal profiles (see Figure 1) that intersect the center of the dose distribution. The measured values shown below were reduced by 2% to correct for the film image and smoothing resolution

**Dose profile direction**	**Isodose width**	**Measurement (mm)**	**Calculation (mm)**	**Difference (mm)**
Left‐Right (L‐R)	50%	5.52 ± 0.08	5.14	0.38 ± 0.08
	25%	9.13 ± 0.13	8.39	0.74 ± 0.13
Superior‐Inferior (S‐I)	50%	5.57 ± 0.08	5.18	0.40 ± 0.08
	25%	9.36 ± 0.13	8.39	0.97 ± 0.13
Anterior‐Posterior (A‐P)	50%	‐	5.40	‐
	25%	‐	8.85	‐

The maximum measured film dose and isodose widths are dependent on the spatial resolution of the film image and the smoothing algorithm in DoseLab. The film was scanned with a resolution of 0.08/mm pixel and the calculated dose was averaged over a radius of 5 pixels to reduce the noise in the film response. To investigate these effects, one of the EBT‐XD films used for patient QA was scanned twice: once using the pixel resolution shown above and then again using a higher resolution of 0.03 mm/pixel. The smoothing algorithm was not applied to the high‐resolution film image. Instead, a Gaussian function was fitted to the two orthogonal dose profiles obtained from this film image for dose points ≥50% of maximum (*r* ^2^ > 0.96). The maximum dose value determined from the two fitted Gaussians was found to be higher compared with the maximum dose obtained from the smoothed, lower resolution image by 0.6%. The full width at half maximum of the Gaussians was found to be lower compared with the 50% isodose width by 2%. These differences were used to correct the maximum dose and the isodose widths obtained from the EBT3 film and are included in the values reported above. An estimated uncertainty for these corrections is included in Table 3.

### 3.2 Output factor constancy

The average output factor for a 2.1‐mm leaf gap, relative to a 10 × 10 cm^2^ field, was 0.0374 with a standard deviation of 0.6%. This was determined from fifteen parallel‐plate chamber measurements over a time period of 6 months. Popple et al.^1^ reported a standard deviation of 0.2%, for the same leaf gap, for nine measurements over a time period of 15 days. Measurements performed with a leaf gap of 2.2, 2.3, and 2.4 mm showed output factors of 0.0382, 0.0390, and 0.0402. These values differ from the average output factor for a 2.1‐mm gap by 2.3%, 4.5%, and 7.6%, respectively. A linear fit to these results shows an output change of 25.1 ± 2.3% per mm relative to a 2.1‐mm gap. One standard deviation in the output factor translates to a leaf gap variation of <0.03 mm. Popple showed a larger sensitivity of 32% per mm, a 3σ difference with the result from this work. This discrepancy is attributed to differences in irradiation geometry; Popple performed these measurements at a larger SDD of 102 cm compared with 100 cm used for this work. The field defined by the 2.1‐mm leaf gap covers a larger volume of the chamber (diameter >> leaf gap) at an SDD of 102 cm, improving the sensitivity to small changes in the leaf gap. While this measurement technique is an excellent tool for detecting small changes in leaf position, the changes in output are dependent on beam and chamber geometry and cannot be directly applied to a virtual cone treatment.

### 3.3 Neutron measurements

The neutron dose for a virtual‐cone treatment designed to deliver 90 Gy was measured as 5.7 ± 0.57 and 5.0 ± 0.5 mSv at distances of 50 and 100 cm from isocenter, respectively. These readings, obtained from a survey meter calibrated in accordance with the US Nuclear Regulatory Commission Regulations,^23^ are assumed to have an uncertainty (*k* = 1) of ±10%. The exposure rate at 50 cm was determined to be less than 3 R/h, well below the gamma rejection threshold of the neutron meter (10 R/h). The difference between the two neutron measurements is much smaller than would be expected from the inverse square; this is attributed to neutron elastic scattering in the treatment room. The measured neutron dose is small but not insignificant, similar to the effective dose from a head CT scan (∼2 mSv).^24^ These results are comparable to the 10‐MV FFF neutron data reported in the literature for Varian Edge and TrueBeam machines. Wen et al.^25^ measured a fast neutron dose (deep‐dose equivalent) of 1.55 × 10^–4^ mSv per MU for a closed field at 50 cm from isocenter using a Luxel+ T series dosimeter. Montgomery et al.^18^ measured an ambient dose equivalent (H*(10)) of 2.86 ± 0.09 × 10^–4^ mSv per MU for a 0.5 × 0.5 cm^2^ field size at a distance of 100 cm from isocenter using a Nested Neutron Spectrometer. Averaging the two measurements from this work and correcting for the change in photo‐neutron dose with depth (Table 1),^19^ yields a deep‐dose equivalent of 1.40 ± 0.14 × 10^–4^ mSv per MU.

### 3.4 TGN treatments

#### 3.4.1 Treatment planning

Table 5 shows the dose to critical structures determined from eight patients previously treated for TGN with a 5‐mm stereotactic cone and then re‐planned using a virtual cone. The calculation time for the virtual‐cone plans was approximately 30 min on a distributed calculation framework comprised of five Eclipse workstations (Windows 10 64‐bit; Intel(R) Xeon(R) CPU E5‐2620 v3, 2.4 GHz). In contrast, the calculation time for the physical‐cone plans was approximately 25 seconds.

**TABLE 5:** Critical structure dose statistics for eight trigeminal neuralgia (TGN) patients (equally split between left and right‐sided conditions) planned using the virtual cone and a physical, 5‐mm stereotactic cone. For each patient, both types of the plan were calculated using the same isocenter and the same dose prescription, 90 Gy to the maximum dose

**Physical cone (5 mm, 6‐MV FFF)**	**Maximum**	**Minimum**	**Mean**	**Standard deviation**	**Planning constraint**
MU	20 668	18 927	19 771	572	‐
Brainstem maximum dose (Gy)	24.97	23.25	24.11	0.55	<25
Brainstem D_0.5cc_ (Gy)	5.54	4.12	4.94	0.44	<10
Brainstem D_0.1cc_ (Gy)	10.23	8.40	9.55	0.67	<12
Brain V_10Gy_ (cc)	1.11	0.73	0.87	0.12	<10
Optic nerve maximum dose (Gy)	0.08	0.01	0.05	0.02	<10
Inner ear maximum dose (Gy)	0.70	0.06	0.24	0.22	<12

**Virtual cone (10‐MV FFF)**	**Maximum**	**Minimum**	**Mean**	**Standard deviation**	**Planning constraint**
MU	31 340	29 300	30 196	602	‐
Brainstem maximum dose (Gy)	22.68	18.75	22.00	1.33	<25
Brainstem D_0.5cc_ (Gy)	6.74	6.33	6.48	0.15	<10
Brainstem D_0.1cc_ (Gy)	11.82	10.58	11.24	0.38	<12
Brain V_10Gy_ (cc)	1.50	1.06	1.24	0.15	<10
Optic nerve maximum Dose (Gy)	1.24	0.54	0.84	0.23	<10
Inner Ear maximum Dose (Gy)	4.10	2.99	3.36	0.36	<12

**FIGURE 2:**
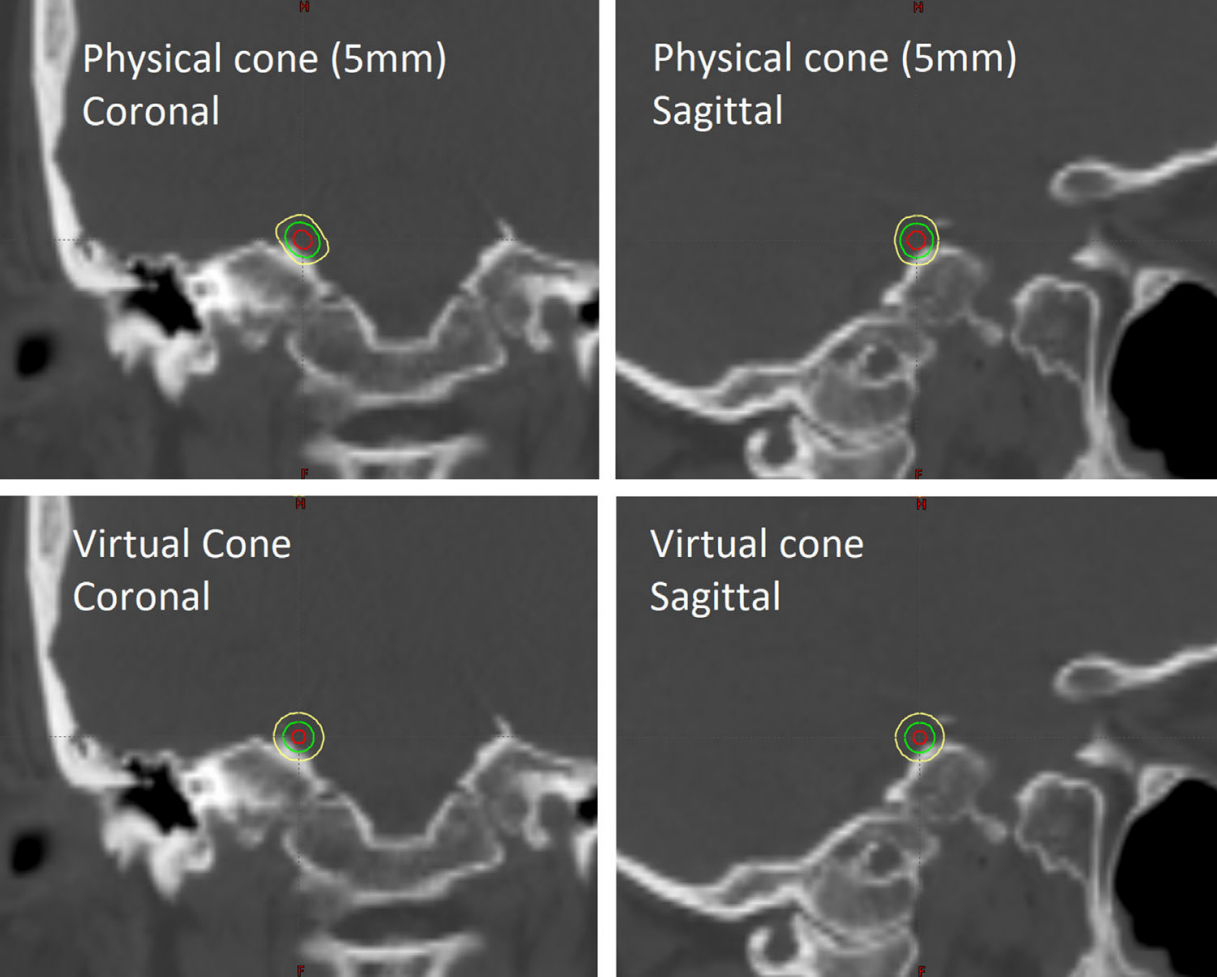
Calculated dose distribution for 5‐mm stereotactic cone and virtual‐cone treatment plans. Both plans were designed to deliver 90 Gy to the root of the right trigeminal nerve. The 80 Gy (red), 45 Gy (green), and 22.5 Gy (yellow) isodose lines are shown

The dose differences between the two types of treatment plans are mostly small. For the same isocenter location and prescription dose, the virtual‐cone plans satisfy all of the planning constraints. The dose to the inner ears and optic nerves is much smaller for the physical cone because the treatment arcs are designed to avoid entrance and exit dose to these structures. The volumetric doses for the brain and the brainstem are slightly larger for the virtual cone; this is attributed to a larger field penumbra associated with an HD120 MLC treatment as well as the greater length and number of arcs used for treatment. Figure 2 shows an example of the dose distribution for both types of treatment plans. The dose distribution for the physical‐cone plan is slightly elongated in the coronal and sagittal planes as a result of the plan geometry discussed previously. Unlike the virtual cone, the physical‐cone plans are not primarily designed to produce a spherical dose distribution.

The biggest difference between the two types of treatment plans is the number of MU. A virtual‐cone plan increases the MU for a TGN treatment by approximately 50%, which significantly increases the photon out‐of‐field dose to the patient. The photon out‐of‐field dose was not measured for this work; however, it has been characterized for an Edge linac by Wen et al. at 10‐MV FFF.^25^ Those measurements indicate that the deep‐dose equivalent, 50 cm from isocenter, would be approximately 13.5 mSv and 20.7 mSv for the mean MU shown in Table 5 for physical and virtual cones, respectively. Although the physical‐cone treatments are performed at 6‐MV FFF, the out‐of‐field dose varies little with beam energy.^19^ If the measured neutron dose from this work is included, the total deep‐dose equivalent at 50 cm is estimated to be 25.3 mSv for a virtual‐cone treatment, versus 13.5 mSv for a physical, stereotactic cone. The dose difference between the two treatment techniques is small but worthy of consideration, particularly for younger patients who are at higher risk of suffering from secondary cancer. Notwithstanding the larger number of MU, the virtual cone treatments are not expected to have a large effect on the linac workload considered for shielding calculations.^26^ TGN treatments are typically performed only one to two times per month on a machine with a patient load of approximately 30 per day. Shielding concerns are further mitigated by the fact that the Edge vault was originally designed to shield beam energies as high as 18 MV.

#### 3.4.2 Patient QA

**FIGURE 3:**
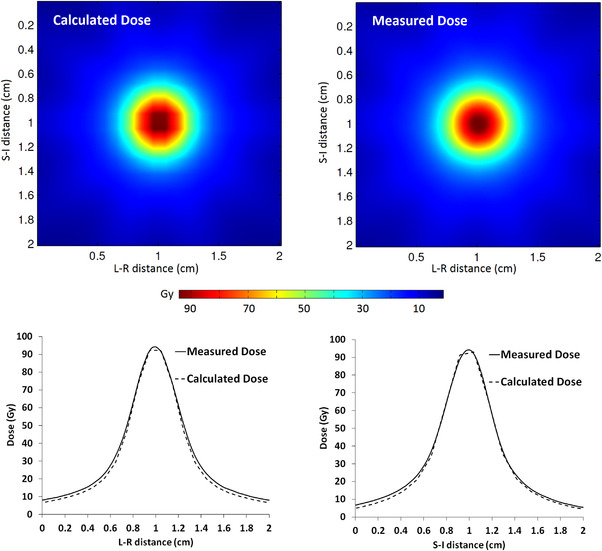
Measured (EBT‐XD film) and calculated (AAA v15.6) dose distributions in the coronal plane for a virtual cone designed for trigeminal neuralgia (TGN) treatment. The dose distributions were registered in DoseLab for gamma analysis using the center of the distributions. The left‐right (L‐R) and superior‐inferior (S‐I) dose profiles are shown for comparison

Figure 3 shows an example of the measured and calculated dose distributions obtained for patient‐specific QA of the seven virtual‐cone TGN treatments. The EBT‐XD film was analyzed in the same way as the EBT3 film: each film image was registered to a DICOM dose plane using the center of the dose distribution and a gamma analysis was applied to a 2 × 2 cm^2^ region of interest centered on the measured dose distribution. All EBT‐XD film measurements for the seven patients showed a gamma passing rate of 100% for an absolute dose difference of 2% and distance‐to‐agreement of 1 mm with a 10% threshold.

Table 6 shows the measured and calculated dose widths in the coronal and sagittal planes for one of the patient plans. A comparison of the dose widths in the coronal plane for all seven patients is shown in Table 7. These results demonstrate an excellent profile constancy and improved agreement over the EBT3 film result. The difference between the measured and calculated maximum dose (in the film plane) varied between ‐6.4 ± 4.0% and 1.9 ± 4.4% across the eight films; the mean difference was ‐2.3 ± 3.1%. The mean result agrees with the measurement‐to‐plan ratio of 1.04 reported by Popple et al.,^1^ to within approximately 2σ, for their QA measurements with EBT‐XD film. The dose widths and the maximum dose differences reported above include a correction for the film resolution and smoothing effects described in Section 3.1.2. These EBT‐XD film measurements, acquired over a time period of 6 months, reinforce the fidelity of treatment demonstrated by the initial W2 and EBT3 film measurements.

**TABLE 6:** Measured (EBT‐XD film) and calculated (AAA v15.6) virtual‐cone dose widths determined for a single patient. Separate quality assurance (QA) plans were developed for the coronal and sagittal plane measurements. The sagittal plane calculation and measurement were performed for a stereotactic radiosurgery (SRS) phantom containing multiple heterogeneity inserts. These inserts were not present for the coronal plane QA, which accounts for the small differences in the calculated superior‐inferior (S‐I) widths between the two planes. The dose widths were determined from orthogonal profiles (see Figure 3) that intersect the center of the dose distribution. The measured values shown below were reduced by 2% to correct for the film image and smoothing resolution

**Coronal plane**				
**Dose profile direction**	**Isodose width**	**Measurement (mm)**	**Calculation (mm)**	**Difference (mm)**
Left‐Right (L‐R)	50%	5.21 ± 0.07	5.06	0.15 ± 0.07
	25%	8.66 ± 0.12	8.31	0.35 ± 0.12
Superior‐Inferior (S‐I)	50%	5.12 ± 0.07	5.28	−0.16 ± 0.07
	25%	8.53 ± 0.12	8.38	0.15 ± 0.12

**Sagittal plane**				
**Dose profile direction**	**Isodose width**	**Measurement (mm)**	**Calculation (mm)**	**Difference (mm)**
Anterior‐Posterior (A‐P)	50%	4.93 ± 0.07	4.91	0.02 ± 0.07
	25%	8.32 ± 0.12	8.08	0.24 ± 0.12
Superior‐Inferior (S‐I)	50%	5.13 ± 0.07	5.12	0.01 ± 0.07
	25%	8.45 ± 0.12	8.24	0.21 ± 0.12

**TABLE 7:** Measured (EBT‐XD film) virtual‐cone dose widths in the coronal plane across seven patients. The dose widths were determined from orthogonal profiles (see Figure 3) that intersect the center of the dose distribution. The measured values shown below were reduced by 2% to correct for the film image and smoothing resolution

**Dose profile direction**	**Isodose width**	**Maximum (mm)**	**Minimum(mm)**	**Mean (mm)**	**Mean difference with calculation (mm)**
Left‐Right (L‐R)	50%	5.21 ± 0.07	5.10 ± 0.07	5.15 ± 0.06	0.13 ± 0.06
	25%	8.66 ± 0.12	8.35 ± 0.12	8.51 ± 0.09	0.24 ± 0.09
Superior‐Inferior (S‐I)	50%	5.18 ± 0.07	5.07 ± 0.07	5.12 ± 0.06	−0.06 ± 0.05
	25%	8.53 ± 0.12	8.30 ± 0.12	8.43 ± 0.09	0.12 ± 0.09

#### 3.4.3 Treatment delivery

The difference in treatment times observed between the virtual and physical cone treatments was small. The average length of time for the seven virtual‐cone treatments from patient time‐out to the termination of the final arc was 48 min; this time includes patient setup, CBCT imaging, and treatment delivery. The average treatment delivery time, the elapsed time between the beginning of the first arc to the termination of the final arc, was 21 min. The corresponding times for the seven most recent TGN patients treated with a physical cone were 46 and 20 min, respectively. Although there are fewer arcs and MU for the physical‐cone treatments, there is a lower maximum dose rate at 6‐MV FFF and the therapists are required to enter the treatment room between each arc to reduce the risk of cone collision during couch rotation.

## 4 CONCLUSIONS

The commissioning results presented in this paper demonstrate that the virtual‐cone dosimetry, first described at UAB, is reproducible on a second Edge linear accelerator at an independent clinical site. This work has built on the UAB data by describing W2 output measurements for a virtual cone, output factor constancy over a sustained period of time, the neutron dose contribution, and a full set of measurement uncertainties for the commissioning data.

The virtual cone is a credible alternative to a physical, stereotactic cone for the treatment of TGN at TCCC. The average difference between the measured and calculated maximum dose determined from the W2 and EBT‐XD film measurements is ‐2.7 ± 1.9% for a 90‐Gy virtual cone. The measured 50% and 25% dose widths were consistently within 0.5 mm of calculation for a 90‐Gy virtual cone. Notwithstanding the uncertainties associated with these measurements, the dose‐effect on the brainstem, located 3–4 mm from isocenter, was limited to <3% of the prescribed dose for a local dose gradient of 10% per mm. There are some higher costs associated with a virtual‐cone treatment, specifically a much longer plan calculation time, additional day‐of‐treatment QA (dose output constancy verification), and a small increase in the out‐of‐field dose to the patient. The larger dose widths seen for the EBT3 film measurement, which indicate the potential for a dose change to the brainstem of >5% of the prescribed dose, also emphasize the continuing need for patient‐specific QA.

## CONFLICT OF INTEREST

The authors declare no conflict of interest for this work.

## AUTHOR CONTRIBUTIONS

All three authors made substantial contributions to this work. Thomas Brown is the primary author and was responsible for writing the manuscript and performing all the data analysis described therein. He was also responsible for designing and performing the neutron dose measurements, as well as collecting dose constancy and patient‐specific‐QA data. Rex Ayers was responsible for building the virtual‐cone beam model and for performing the initial output and profile (W2 detector and EBT3 film) measurements. He was also responsible for collecting dose constancy and patient‐specific QA data. Richard Popple was responsible for the design of the initial output and profile measurements and the design of the dose constancy measurements. He provided directions for building the virtual‐cone beam model, using the virtual‐cone planning script, and performing pre‐treatment MLC QA. Rex Ayers and Richard Popple have reviewed the manuscript and provided edits prior to submission.

## Data Availability

Research data are not shared.
